# Synergistic effects of organic fertilizer and corn straw on microorganisms of pepper continuous cropping soil in China

**DOI:** 10.1080/21655979.2020.1840753

**Published:** 2020-10-30

**Authors:** Jingxia Gao, Hongxia Pei, Hua Xie

**Affiliations:** Institute of Germplasm Resources, Ningxia Academy of Agriculture and Forestry Sciences, Yinchuan, Ningxia, China

**Keywords:** Pepper, continuous cropping, organic fertilizer, corn straw, microbial community

## Abstract

Because of the large population, large demand, limited arable land and many environmental factors, continuous cropping have become a very common phenomenon in China. However, long-term continuous cropping has caused a series of serious soil-borne diseases, and the yield and quality of crops to drop, which seriously restricted the sustainable development of agricultural industry. Therefore, in order to improve the yield of pepper and reduce the occurrence of soil-borne diseases, it is essential to understand the effect of continuous cropping of pepper on soil microbial community composition and abundance. In this study, high throughput sequencing was used to study the effects of seven treatments of organic fertilizers and corn straw on soil microbial community and function of pepper continuous cropping. The results showed that the yield of all treatments was significantly higher than that of the control. The soil microbial diversity and community composition showed that *Proteobacteria* and *Ascomycota* were the most abundant phylum in all treatments. In conclusion, there were significant differences among the seven treatments and the treatment of fowl dung with corn straw was the best fertilizer combination to improve the yield and output value of pepper. Besides, the addition of fowl dung and corn straw not only can improve the community and functions of microorganisms, but also enhance the ability of disease resistance, and ultimately decrease the soil-borne diseases. The results will help to provide scientific basis for rational application of organic fertilizer and corn straw, and overcoming continuous cropping obstacles.

## 1. Introduction

**Figure:**
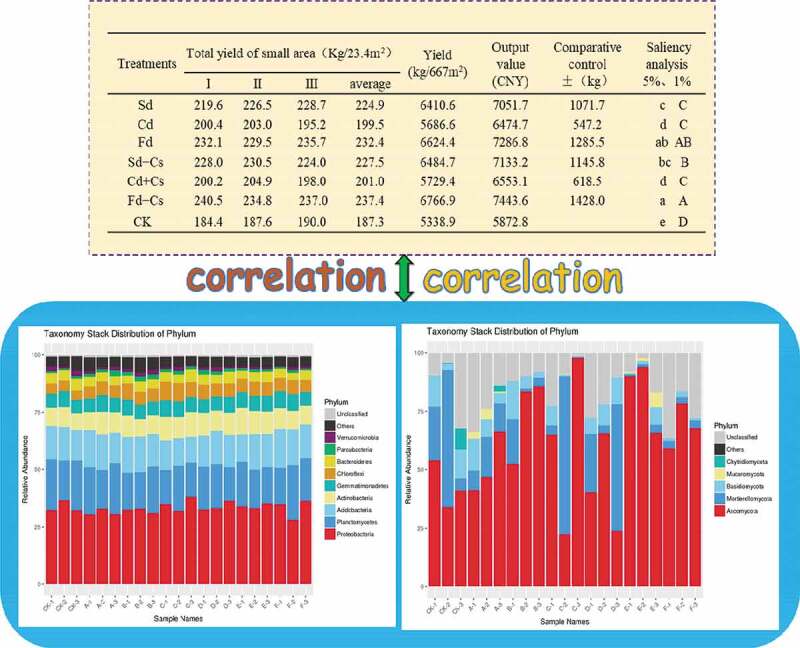

Pepper, one of the most important essential crops, has a large planting area with high nutritive value, such as rich polyphenols, capsaicin, ascorbic acid, plant chemicals and so on [1]. Among all province of China, Ningxia is very suitable for planting of pepper because of the compatible annual average temperature, frost-free period and the precipitation. Due to production demand, limited arable land and many environmental factors, continuous cropping is common phenomenon in this area [2,3]. However, long-term continuous cropping will cause the reduction of soil fertility and microbial diversity, increase of serious soil-borne disease obstacles [4–6], and decline of yield and quality decline [7], which restrict the sustainable development of pepper industry severely [8–11].

In recent years, a series of problems, such as insufficient soil fertility, crop yield decline and environmental pollution caused by continuous cropping, have been improved by the application of organic fertilizer and returning straw to the field. Increasing the application of organic fertilizer and straw returning can not only provide comprehensive nutrients for crops [12], but also significantly improve the soil physical and chemical properties, increase the content of available nutrients in the soil, while enhancing the ability of water and fertilizer conservation of crops and improve the diversity of soil microbial community, which can provide good soil environment for crops, and improve the quality of crops [13–15]. In recent years, more and more studies have speculated that the destruction of soil microbial community is also one of the reasons for continuous cropping obstacle after long-term continuous cropping [4,16,17]. Soil microorganism is an important part of soil ecosystem, which plays an important role in nutrient cycling, bioremediation, soil organic matter stability and soil aggregate formation [18–20]. Application of organic fertilizer can significantly increase the number and activity of soil microorganisms, and then directly improve the structure and increase functions of soil microbial communities [21]. Zhou et al. showed that the abundance and diversity of antibiotic resistance genes (ARGs) were the lowest in earthworm organic fertilizer, and the highest in chicken manure organic fertilizer. In addition to increasing the availability of soil organic matter and nutrients, organic fertilizer can also promote the activities of microorganisms as a biologically active agent and/or enhance the synergistic interaction among soil microbial groups, thus further increasing plant biomass [22]. However, straw returning can not only affect the growth and reproduction of microorganisms through changing the physical and chemical properties of the main soil, but also provide a rich material basis for the growth and reproduction of microorganisms, thus promoting the increase of the number of soil microorganisms [23]. Huang et al. found that cotton straw significantly increased soil organic matter, available nitrogen, available phosphorus and available potassium, and promoted the growth of cucumber seedlings. Additionally, the concentration of cotton straw was positively correlated with the number of soil culturable microorganisms and the total soil microbial biomass [24]. Lu et al. drew the conclusion that the application of corn straw had a positive impact on soil properties and soil microbial communities [23].

Although the effects of organic fertilizer and straw on soil microorganisms have been studied, that of the synergistic effect between them is not clear enough. Thus, in this study, the effects of organic fertilizer and straw returning on soil microbial community and function in continuous cropping of pepper were studied by analyzing the richness and diversity of 16S rRNA gene V3-V4 region of soil bacteria and ITS2 region of fungi after DNA extraction, PCR amplification and database construction through high throughput sequencing. Our research will provide theoretical basis for rationally applying organic fertilizer and straw, and overcoming the obstacles of continuous cropping.

## 2. Materials and methods

### 2.1. Experimental sites and design

The experiment was carried out in pepper demonstration base (35°45ʹN-36°14ʹN, 106°52ʹE-106°21ʹE), Xinji Township, Pengyang County, Ningxia, China (continuous cropping for 5 years). The annual temperature, frost-free period and total precipitation in this region are 7.5°C, 158 d and 442.7 mm, respectively. The soil type is yellow loam and the physical and chemical properties of soil as following: total nitrogen1.07 g·kg^–1^; total phosphorus 0.95 g·kg^–1^; total potassium 22.00 g·kg^–1^; organic matter 24.00 g·kg^–1^; alkali-hydrolyzed nitrogen 63.00 g·kg^–1^; available phosphorus 29.40 g·kg^–1^, available potassium 192.00 g·kg^–1^, pH 7.5, and total salt 1.10 g·kg^–1^.

The field experiment was carried out in an arched shed and the organic fertilizer was decomposed before the experiment. The material we used in the experiment is Niujiao pepper, and the variety is Jufeng No. 1, which was provided by Ningxia Jufeng Seedling Co., Ltd. On 27 March 2017, the organic fertilizer and corn stalks were applied to the arch shed in accordance with the experiment design. The experiment was divided into seven treatments, including: (A) cow dung 5000 kg·667 m^−2^ and corn straw 2000 kg·667 m^−2^, (B) fowl dung 1500 kg·667 m^−2^ and corn straw 2000 kg·667 m^−2^, (C) sheep dung 4000 kg·667 m^−2^ and corn straw 2000 kg·667 m^−2^, (D) fowl dung 1500 kg·667 m^−2^, (E) cow dung 5000 kg·667 m^−2^, (F) sheep dung 4000 kg·667 m^−2,^ and control (CK) with no organic fertilizer. The detailed descriptions of the treatments are shown in Table 1.

**Table 1.** Experimental treatment details. Cd+ Cs: cow dung 5000 kg·667 m^−2^ and corn straw 2000 kg·667 m^−2^, Fd+Cs: fowl dung 1500 kg·667 m^−2^ and corn straw 2000 kg·667 m^−2^, Sd+Cs: sheep dung 4000 kg·667 m^−2^ and corn straw 2000 kg·667 m^−2^, Fd: fowl dung 1500 kg·667 m^−2^, Cd: cow dung 5000 kg·667 m^−2^, Sd: sheep dung 4000 kg·667 m^−2^, CK: no organic fertilizer

Treatments	Fertilizer dosage (kg/m^2^)	Fertilizer category
Cd+ Cs	5000/667	Cow dung
	2000/667	Corn straw
Fd+Cs	1500/667	Fowl dung
	2000/667	Corn straw
Sd+Cs	4000/667	Sheep dung
	2000/667	Corn straw
Cd	5000/667	Cow dung
	/	/
Fd	1500/667	Fowl dung
	/	/
Sd	4000/667	Sheep dung
	/	/
CK	/	/
	/	/

### 2.2. Soil sampling

The samples from the top 30-cm of the pepper rhizosphere soil were collected according to five sampling points using the ‘Z’ shape. All soil samples were collected using sterile containers and then transported on dry-ice to the laboratory, then samples were stored at −80°C for DNA extraction.

### 2.3. DNA extraction, amplification, and sequencing

DNA from 500 mg soil sample was extracted using CTAB method [25]. NanoDrop Spectrophotometer (Thermo Fisher Scientiﬁc Inc., United States) was used for determining the quality and concentration of extracts. The 16S rRNA gene in V3-V4 region and the fungal gene in ITS2 region were amplified by the primers 341F [5ڹ-CCTACGGGNGGCWGCAG-3ʹ] and 806R [5ʹ-GGACTACHVGGGTATCTAAT-3ʹ], and KYO2F [5ʹ-GATGAAGAACGYAGYRAA-ʹ]and ITS4RT [5ʹ-CCTCCGCTTATT GATATGC-3ʹ], respectively. All PCR reactions were carried out with 15µL of Phusion® High-Fidelity PCR Master Mix (New England Biolabs), 0.2 µM of forward and reverse primers and approximately 10 ng template DNA. Specific reaction procedures included initial denaturation at 98°C for 1min, followed by 30 cycles of denaturation at 98°C for 10 s, annealing at 50°C for 30 s, elongation at 72°C for 30 s, and finally 72°C for 5 min. Then, PCR production was detected by 2% agarose gel, which was purified with Qiagen Gel Extraction Kit (Qiagen, Germany). Sequencing libraries were constructed by usingTruSeq® DNA PCR-Free Sample Preparation Kit (Illumina, USA) according to manufacturer’s instructions. The Qubit 2.0 Fluorometer (Thermo Scientific) and Agilent Bioanalyzer 2100 system were used to evaluate whether the sequencing library is qualified. High-throughput sequencing of the bacterial 16S rRNA and fungus ITS2 gene were performed on Illumina HiSeq2500 platform by Gene-Denovo Bioinformatics Technology Co. Ltd (Guangzhou, China). The raw reads were deposited into the NCBI Sequence Read Archive (SRA) database (Accession Number: SRP256106 and SPR111).

### 2.4. Data and statistics analysis

All statistical differences were calculated by ANOVA, and the mean was analyzed by LSD multiple comparison test using IBM SPSS statistical software (SPSS, v.19), *P* < 0.05 was considered to be significantly different. The diversity indices of Shannon and Simpson and richness indices of Chao1 and ACE were calculated using QIIME software (version 1.7.0) [26].

## 3. Results

### 3.1. Pepper yield analysis

The average values of pepper yield and output value in three years showed that six treatments were significantly significant compared with the control at 5% and 1% levels (Table 2). In the treatment of fowl dung and fowl dung with corn straw, cow dung and cow dung with corn straw, sheep dung and sheep dung with corn straw, there were no significant at 5% level. There were significant differences among treatments of cow dung, sheep dung and fowl dung, but there was no significant difference between cow dung and sheep dung at 1% level. In conclusion, six kinds of combined fertilization can effectively improve the yield and output value of pepper, and the treatment of interaction between chicken manure and corn straw was the best fertilizer combination to improve the yield and output value of pepper compared with other treatments.

**Table 2.** The yield of pepper analysis under each treatment in three years

Treatments	Total yield of small area (Kg/23.4 m^2^)				Yield (kg/667 m^2^)	Output value (CNY)	Comparative control ± (kg)	
								Saliency analysis
	I	II	III	Average				5%, 1%
Sd	219.6	226.5	228.7	224.9	6410.6	7051.7	1071.7	cC
Cd	200.4	203.0	195.2	199.5	5686.6	6474.7	547.2	dC
Fd	232.1	229.5	235.7	232.4	6624.4	7286.8	1285.5	ab AB
Sd+Cs	228.0	230.5	224.0	227.5	6484.7	7133.2	1145.8	bc B
Cd+Cs	200.2	204.9	198.0	201.0	5729.4	6553.1	618.5	dC
Fd+Cs	240.5	234.8	237.0	237.4	6766.9	7443.6	1428.0	aA
CK	184.4	187.6	190.0	187.3	5338.9	5872.8		e D

† Significant under 5% and 1% level labeled a, b, c, d and A, B, C, D, respectively.

### 3.2. Diversity and composition of soil bacterial and fungal communities

**Figure 1.**
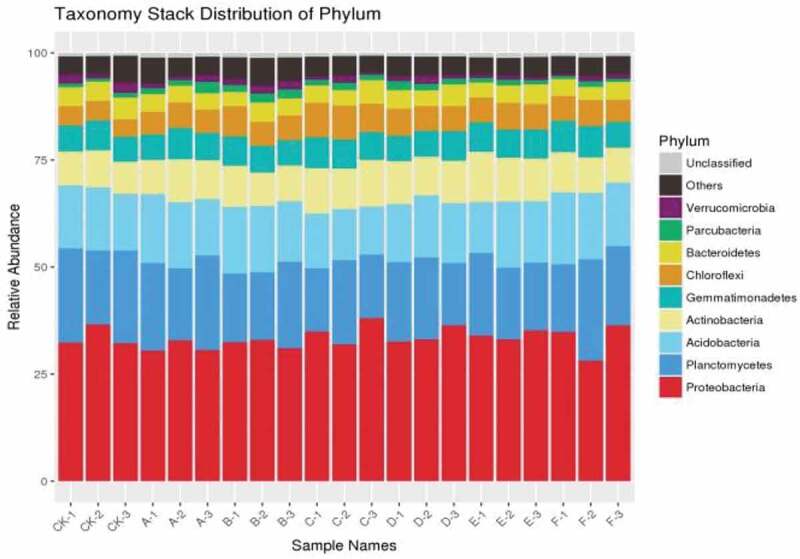
The relative abundance of soil bacterial in each samples at phylum level

The number of operational taxonomic units (OTUs) (at a 97% similarity level) in 7 treatments was between 55,336 and 99,329 (Table 3). The index values of OTUs, ACE and Chao 1 showed that there was a similar trend among the seven treatments and there was significant difference between CK and other treatments. For Shannon index, there was no significant difference among Sd, Fd + Cs and CK and Simpson diversity index showed that Cd, Fd and CK had significant difference at 5% level. There were nine phylums, of which the relative abundances were above 2% (Figure 1). Interestingly, *Proteobacteria* and *Planctomycetes* were the most abundance phylum in the seven treatments. There were significant differences among other treatments compared with the control, which indicated a similar trend in richness and diversity (Chao1 and ACE). There was no significant difference in Shannon diversity between sheep dung (F) treatment and control group. Compared with the control, there were significant differences in cow dung, chicken dung, cow dung, cow dung with cow dung, and cow dung with cow dung. It was seen that there were significant differences between cow dung and CK, as well as between fowl dung and CK from Simpson index (Table 3). The alpha diversity of fungi is shown in Table 4. At 97% similarity level, the number of OTU in 7 treatments ranged from 88,613.667 to 105,448.667. There was no difference in the number of OTUs between treatment groups. The diversity and richness of fungi were calculated in seven treatments by Chao1 and ACE. The results suggested Fd had significant difference compared with the treatment of Fd + Cs at 5% level. In terms of Shannon index, the difference between the treatments of Sd and Fd + Cs, and between Cd and Fd + Cs, were analyzed and a conclusion was drawn that there was no difference between Sd and Cd. Additionally, we found there existed the differences among Sd, Cd, CK and Fd + Cs, respectively (Table 4).

**Table 3.** Alpha diversity of the 16S rRNA gene seven treatments

Treatment	OTUs	Chao1	ACE	Shannon	Simpson
Sd	97,655 ± 6876.898b	6560.579 ± 192.0780b	6567.663 ± 375.558b	10.279 ± 0.076a	0.997 ± 0.00039a
Cd	94,550 ± 16,252.092b	6514.343 ± 25.127b	6414.842 ± 72.141b	10.461 ± 0.090b	0.998 ± 0.00032b
Fd	99,329 ± 3818.049b	7310.679 ± 88.269b	7324.22 ± 207.751b	10.663 ± 0.089b	0.998 ± 0.00035b
Sd+Cs	92,330 ± 4050.551b	7030.774 ± 41.844b	7053.968 ± 60.868b	10.36 ± 0.121b	0.998 ± 0.00033a
Cd+Cs	87,677 ± 9267.735b	6837.624 ± 115.852b	6812.933 ± 171.164b	10.47 ± 0.102b	0.998 ± 0.00018a
Fd+Cs	82,214 ± 14,919.157b	6789.442 ± 236.232b	6762.135 ± 232.413b	10.244 ± 0.054a	0.997 ± 0.00009a
Ck	55,336 ± 22,734.977a	5856.616 ± 410.120a	5903.657 ± 369.507a	10.189 ± 0.103a	0.997 ± 0.00025a

† Different letters in the same column represent significant differences (*P*< 0.05) Values are means±standard deviation (n = 3).

**Table 4.** Alpha diversity of fungi in seven treatments

Treatment	Chao1	Ace	Shannon	Simpson
Sd	88,613.667 ± 581.085	224.101 ± 10.213ab	4.623 ± 0.828b	0.902 ± 0.088b
Cd	98,553 ± 8469.918	222.443 ± 10.250ab	4.936 ± 0.145b	0.945 ± 0.010b
Fd	102,510 ± 12,155.308	216.658 ± 4.691b	4.290 ± 0.775ab	0.848 ± 0.120ab
Sd+Cs	90,915.667 ± 11,748.480	221.020 ± 23.829ab	3.919 ± 1.515ab	0.782 ± 0.209ab
Cd+Cs	87,728.333 ± 12,840.783	225.567 ± 24.265ab	4.260 ± 0.868ab	0.860 ± 0.109ab
Fd+Cs	105,448.667 ± 11,472.812	248.631 ± 21.884a	3.034 ± 1.099a	0.644 ± 0.199a
Ck	104,939.667 ± 8456.383	229.647 ± 8.543ab	4.523 ± 0.350ab	0.911 ± 0.024b

† Different letters in the same column represent significant differences (P < 0.05). Values are means ± standard deviation (n = 3).

**Figure 2.**
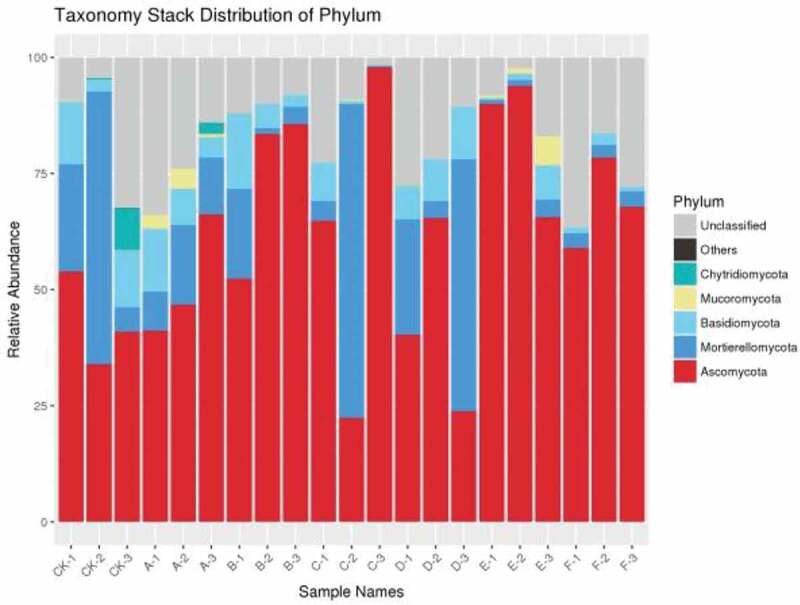
The relative abundance of soil fungus in each samples at phylum level

The relative abundance of five phylums was higher than 2% and *Ascomycetes* and *Mortierellomycota* were the top most phylums among the seven treatments. Compared with the control, the relative abundance of *Ascomycetes* in other treatments was significantly different, especially in E (cow dung) and F (sheep dung) treatments, while the relative abundance of *Mortierella* and *Basidiomycetes* in E (cow dung) and F (sheep manure) treatments were lower than that other treatments. Whereas, *Mucoromycota* was only found in treatments of cow dung with corn straw (Cd + Cs) and cow dung (Cd), and Aspergillus was detected in some samples of CK-3 and A-3, but not in other samples of the same group. What’ s more, the existence of *Chytriomycota* was further verified. In a word, different fertilization methods can change the abundance and composition of the microbial community of pepper soil fungi, which make it more suitable for the growth and development of pepper, and reduce the soil-borne diseases caused by long-term continuous cropping activities (Figure 2).

### 3.3. Fungal functional gene analysis

**Figure 3.**
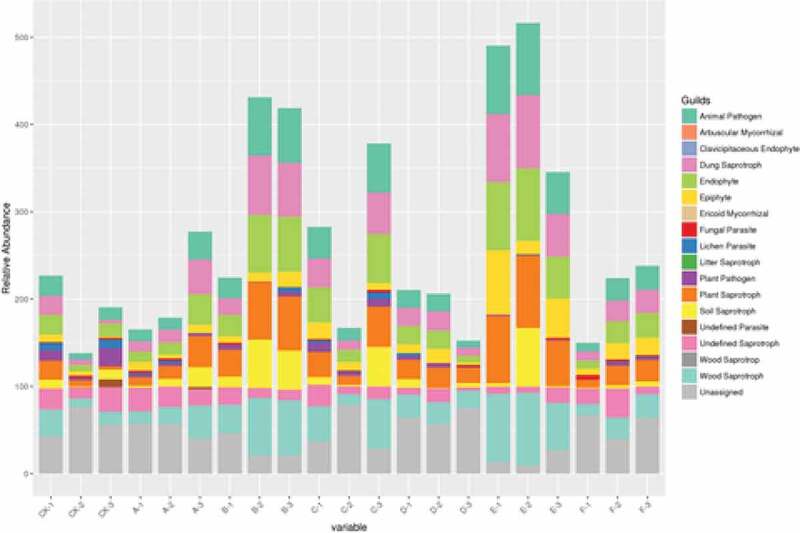
The function predictive analysis of fungi

Based on the abundance of OTUs, FUNGuild was used to perform functional annotation of fungi. The relative abundance of animal pathogens, fecal saprophytic bacteria, endophytes, fungi, plant saprophytic bacteria, soil saprophytic bacteria and wood saprophytic bacteria in chicken dung treated with corn straw (FD + CS) were significantly different from those of other treatments. In this study, we found that the relative abundance of lichen parasites significantly decreased in some treatments, especially in cow dung (CD) and sheep dung (CD) (Figure 3). Compared with the traditional fertilization, organic fertilizer combined with base fertilizer can reduce the damage to fungi and improve the yield of pepper, which will be suitable for the growth of pepper.

### 3.4. Relationships between bacterial and fungus community structure and the total yield of pepper

**Figure 4.**
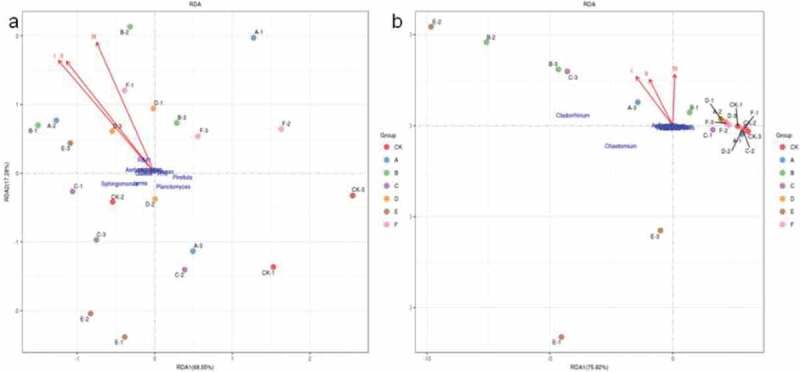
RDA analysis between bacterial and fungal community structure, and the total yield of pepper. (a) the correlations between bacterial communities and the total yield of pepper and (b) the correlations between fungal communities and the total yield of pepper. I, II and III labeled for the total yield of pepper three years. The direction of an arrow indicates the steepest increase in the variable and the length indicates the strength relative to the other variables

Redundancy analysis (RDA) was used to analyze the relationship between the community structure of soil bacteria and fungi and the total yield of pepper (Figure 4). As shown in Figure 4(a), 68.55% of the variation can be explained by the relationship between bacteria and pepper yield, while 75.92% variation between fungi and pepper yield (Figure 4(b)). The results showed that the correlation between the bacterial and fungal community structure, and the total yield of pepper had a similar trend. There was a significant negative correlation between the three yields of pepper and *Sphingomonas, Planctomyces, Pirellula, Lysobacter, RB41, H16 and Iamiac*. It was seen that *Cladorrhinum* and *Chaetomium* had closely negative correlation with I and II years yield of pepper, while closely positive correlation with III yield of pepper.

## 4. Discussion

Understanding the effects of organic fertilizer and straw on the diversity and composition structures of soil bacteria and fungi under continuous cropping system of pepper will help to improve soil productivity under long-term continuous cropping. Long-term continuous cropping leads to the decrease of organic matter content, serious soil diseases, and the decline of yield and quality due to the unscientific fertilization and cultivation system. Compared with our results, previous studies have reported that continuous cropping caused the decrease of soil organic matter and increase of serious soil-borne diseases [27]. With the increase of pepper planting years, soil organic matter increased significantly, which was also related to the long-term oversupply of chemical fertilizer [4]. However, the diversity and composition structure of soil bacterial and fungal communities are considered to be the key factors affecting soil quality. In our study, the diversity of soil bacteria and fungi community decreased significantly with the pepper cultivation for three years. The results showed that the raise of the soil organic matter content and scientific fertilization could help to increase the abundance of soil microbial community composition in pepper. Interestingly, the long-term continuous cropping of pepper was suitable for planting in Ningxia Hui Autonomous Region because of the yellow soil type.

The results showed that there were significant differences in pepper yield between seven treatments and control at 5% and 1% levels (Table 2). Compared with the previous studies on black pepper [16], cotton [28], watermelon [9], cucumber [29], the growth of pepper crop has a significant impact on microbial community diversity after continuous cropping. In our study, the diversity and communities structure of soil bacterial and fungal showed that *Proteobacteria* and *Ascomycetes* had the highest abundance at the phylum level, which was basically consistent with the previous views that *Proteobacteria* was the most common phylum [30,31]. The relative abundance of *Ascomycetes* of soil fungi was significant at the phylum level, which may be caused by the long-term continuous cropping system. With the long-term continuous cropping of pepper, the relative abundance of *Ascomycota* increased, which was is similar to previous studies [32–35]. Additionally, previous studies also showed that *Proteobacteria* and *Ascomycota* were the most abundant in continuous cropping [16,28,36–38]. In a word, continuous cropping of pepper can cause the increase of serious soil-borne diseases, and the decrease of yield and quality. As the study found that pathogenic fungi may be the main reason for the soil-borne diseases of pepper, which could invade the tissues of plants [39]. By adding the fowl dung and corn straw, the relative abundance of microorganism in the soil of pepper could be increased, decrease the amount of pathogenic fungi, and finally decrease the occurrence of soil-borne diseases. Through the analysis of the bacterial and fungal communities of pepper, the diversity of soil fungi was revealed, which was significantly affected by the continuous cropping system of pepper. These results may lead to the decline of pepper yield and eventually caused serious soil-borne diseases. Therefore, an appropriate fertilization and tillage system is a great one way to improve soil-borne diseases caused by long-term continuous cropping.

## 5. Conclusion

By analyzing the seven treatments in the study, there were significant differences among cow dung, sheep dung and fowl dung treatment, except the treatment of cow dung and sheep dung at 1% level. The treatments of fowl dung with corn straw were a best choice for increasing pepper production and output value, and reducing soil-borne disease obstacles. This treatment can improve the abundance and composition of fungal community and microbial community, which is more suitable for the growth and development of pepper and reduction of the soil-borne diseases caused by long-term continuous cropping.

## Highlights

All treatments were higher than that of the control for the yield of pepper.

The treatment of fowl dung with corn straw was the best choice for pepper production.

The treatment of fowl dung with corn straw can improve the microbial community.
